# The effect of the surgical treatment of brachycephalic obstructive airway syndrome on the thermoregulatory response to exercise in French bulldogs: a pilot study

**DOI:** 10.3389/fvets.2023.1229687

**Published:** 2023-10-12

**Authors:** Žiga Žgank, Alenka Nemec Svete, Helena Lenasi, Janez Vodičar, Vladimira Erjavec

**Affiliations:** ^1^Small Animal Clinic, Veterinary Faculty, University of Ljubljana, Ljubljana, Slovenia; ^2^Faculty of Medicine, Institute of Physiology, University of Ljubljana, Ljubljana, Slovenia; ^3^Faculty of Sports, Institute of Sport, University of Ljubljana, Ljubljana, Slovenia

**Keywords:** brachycephalic dog, BOAS, thermoregulation, temperature, treadmill, exercise test, folded flap palatoplasty, vertical wedge rhinoplasty

## Abstract

**Introduction:**

Due to altered anatomy of the upper respiratory tract, brachycephalic dogs exhibit increased airway resistance and reduced surface area for evaporative heat loss, predisposing them to respiratory and thermoregulatory problems, a syndrome referred to as Brachycephalic Obstructive Airway Syndrome (BOAS). Compared to non-brachycephalic dogs, brachycephalic breeds are more susceptible to heat-related injuries even at low ambient temperatures and relatively low physical activity levels. Surgical treatment alleviates clinical signs, potentially improving dogs’ thermoregulatory ability with BOAS. Our study aimed to investigate the thermoregulatory response in French bulldogs before and after the surgical correction of BOAS, respectively.

**Methods:**

Thirteen dogs were exposed to dynamic exercise on a treadmill and the dynamics of their rectal temperature (RT) and heart rate (HR) was measured. The experiment was performed in two independent sessions, before and after the surgical treatment. The test consisted of two consecutive 5-min walks at a speed of 2.5 km/h, first at an inclination of 0% and the second at an inclination of 5%, and a 30-min recovery period. Rectal temperature and HR were measured before the start of the test (t0), at the end of the first (t1) and the second part (t2), and 15 min (t3) and 30 min (t4) in the recovery.

**Results:**

A significant increase in RT and HR was observed during exercise and recovery compared to the resting values, implying that the exercise intensity was sufficient to challenge the thermoregulatory response. The increase of RT was significantly lower during both parts (t1: *p*  = 0.004; t2: *p*  <  0.001) of exercise after the surgical treatment than before the treatment. Although a trend of lower RT after recovery was observed compared to before the surgery, it did not reach statistical significance. Similarly to RT, the HR was significantly lower during exercise after the first (*p*  =  0.020) and the second part (*p*  =  0.011) of exercise after the surgery compared to before the surgery but did not reach significance in the recovery.

**Conclusion:**

Surgical treatment of BOAS can improve thermoregulation during exercise in French bulldogs with BOAS.

## 1. Introduction

The growing popularity and years of selective breeding of short-muzzled dog breeds have resulted in an increased number of brachycephalic dogs with extreme confirmation morphology (1–4). Standardized conformational measurements of craniofacial ratio, eye width ratio, skull index, neck girth ratio, and neck length ratio can help identify affected dogs (4). The most extreme brachycephalic breeds with the most exaggerated phenotypic traits are French and English bulldogs, pugs (1–3, 5–8), and Boston terriers (9). Extreme brachycephalic breeds are 15 times more likely to have an insurance claim submitted because of problems related to brachycephaly, and the majority of affected dogs receive the diagnosis before they are 2 years old (9, 10). Their congenitally shortened and wide skulls cause compression of soft tissues of the upper airway, which are not reduced in proportion to the skeletal changes (1, 3, 11). These soft tissue changes include stenotic nares, an elongated and thickened soft palate, everted saccules on the lateral side of the vestibular folds of the larynx, narrowed rima glottidis, and collapse of the larynx and trachea (2, 3, 12). Compression of the nasal passages leads to abnormal configuration of the rostral aberrant turbinates and nasopharyngeal turbinates (caudal turbinates) which are aberrant and protrude beyond their normal anatomical limits, resulting in the obstruction of the nasal and/or nasopharyngeal meatus (1, 13, 14). The nasopharynx is further narrowed by a dorsally displaced soft palate due to a thick tongue, which at the same time also closes the oropharynx (15). The narrowing caused by soft tissue results in partial or complete obstruction of airflow through the upper airway, leading to increased airway resistance and subsequently severe respiratory distress, as well as problems with thermoregulation (2, 3, 16). This conformation-related disorder is referred to as Brachycephalic Obstructive Airway Syndrome (BOAS) (3, 4, 11). Typical clinical signs include snoring, inspiratory dyspnea, stridor, cyanosis, sleep apnea, exercise and heat intolerance, and even syncopal episodes in more severely affected animals. Increased ambient temperatures and humidity, excitement, exercise, and stress can worsen these signs (1, 5, 17).

The most effective mechanism of heat elimination in dogs is evaporation through panting and respiration. Panting allows large amounts of air to be in contact with the moist mucosal membranes of the nasal turbinates, oral cavity, and tongue that provide evaporative surfaces. When exposed to exercise or high environmental temperatures, ventilation occurs through the mouth and nose, and heat loss is additionally achieved by panting (18, 19). However, even panting can become inefficient during exercise, and when the relative humidity rises (>80%), in these settings, heat dissipation fails, and the body starts to accumulate heat (18–23). Relatively low environmental temperatures (around 23°C) might render thermoregulatory mechanisms ineffective even in non-brachycephalic dogs suffering from obesity, cardiovascular or respiratory problems (20, 22). In the study of Bruchim et al. (24), temperatures at least 24.1°C were sufficient to induce heat-related injuries (HRI) in the majority of dogs included in the study. Moreover, in the study of Hall et al. (25), the median temperature that caused exertional HRI was as low as 16.5°C.

In mongrel dogs, nasal resistance accounts for 76.5% of total airflow resistance (26). Upper airway resistance to flow is much greater in brachycephalic dogs with stenotic nostrils, aberrant conchae, mucosal hypertrophy of the turbinates, and mucosal contact points within the nasal cavity than in non-brachycephalic breeds and increases to 80% (14, 27, 28). Because of the increased resistance during inspiration which is similar to humans with nasal obstruction secondary to complex nasal defect (29), dogs must produce higher negative intrapleural pressure to overcome increased resistance (12, 30). The higher effort to increase respiratory volume imposes an additional burden on the respiratory muscles, increasing their work and thus producing metabolic heat (31). In addition, evaporative heat dissipation through panting is impaired in brachycephalic dogs due to the greatly reduced surface area; therefore, brachycephalic breeds are less able to effectively dissipate heat during heat stress (16), which is additionally hampered during exercise and can lead to hyperthermia (12, 20, 24). One third of brachycephalic dogs reportedly have problems with thermoregulation (23, 32), and due to their impaired thermoregulatory capabilities, brachycephalic breeds (English bulldogs, French bulldogs, pugs) are 2.1-times more susceptible to HRI even when ambient temperatures are relatively low or at relatively low physical activity or exercise (33). Moreover, brachycephalic dogs have a three-fold higher risk of fatal outcomes after HRI (20, 24, 25, 33).

The aim of BOAS treatment is to reduce airway obstruction, consequently improving the quality of life of affected dogs (30). This can be achieved by surgical treatment, which comprises the correction of stenotic nares, elongated and thickened soft palate, laryngeal saccules in selected cases and Laser- Assisted Turbinectomy (LATE) (5, 30, 34–36). The goal of surgical correction is to reduce airway resistance, which can improve clinical signs in the short term and prevent worsening or even reverse secondary soft tissue changes (2, 12, 30, 37).

To the best of our knowledge, no data are available regarding how surgical treatment of BOAS affects body temperature regulation in dogs with BOAS.

Accordingly, our study aimed to evaluate the thermoregulatory response in French bulldogs, representatives of an extreme brachycephalic breed, exposed to dynamic exercise on a treadmill before and after surgical treatment of BOAS. To this end, their rectal temperature (RT) was tracked before, during and at different time points after the cessation of exercise in two independent sessions, i. e. before and after the surgical treatment. It was hypothesized that the increase in body temperature of French bulldogs during exercise would be lower after the surgical procedure and that their body temperature would return to normal in recovery in a shorter time than before the surgery.

## 2. Materials and methods

### 2.1. Study animals

The study was conducted at the Small Animal Clinic of the Veterinary Faculty, University of Ljubljana, Slovenia, and in the Laboratory of Physiology of the Faculty of Sports, University of Ljubljana, Slovenia, from 2021 to 2022. Fourteen privately owned French bulldogs were recruited, of which 13 were eventually included in the study. One dog was excluded because it could not complete the exercise test (ET). The inclusion criteria were that there were no coexisting systemic diseases, the dogs had not been medically treated or vaccinated within the previous month, were diagnosed with BOAS, and had not had previous upper airway surgery. The diagnosis of BOAS was based on clinical signs and clinical examination and confirmed with an endoscopic examination of the upper airways. All dogs included in the study presented with severe multilayer upper airway obstruction and were classified as BOAS+ (38). Formal written informed consent was obtained from the owners before the dogs participated in the study. All procedures in the study complied with the relevant Slovenian government regulations (Animal Protection Act, Official Gazette of the Republic of Slovenia, No. 43/2007). The study was approved by the Animals in Experiments Welfare Commission of the Veterinary Faculty, University of Ljubljana, approval number 18-3/2022-1.

Each dog underwent two independent ET before and after the surgery. Before the start of the ET, a physical examination was performed, and the body condition score (BCS) assessed using a five-point BCS chart (39). Clinical examination performed before ET revealed moderate to severe respiratory noises (stridor, stertor), increased respiratory effort, and abdominal breathing. The owners completed a questionnaire asking about the dog’s breathing problems, general health, and fitness status, which had to be completed two times: before and six (to nine) months after the surgery when the second setting of ET was applied. The questionnaire, using a five-point score, was adapted from Erjavec et al. (36), grading each question from 1 to 5 (“1” meaning that the clinical sign was not present and “5” meaning that the clinical sign was always present). The questionnaire is in the appendix.

### 2.2. Exercise test

Under controlled conditions, the exercise test was performed in the Laboratory of Physiology of the Faculty of Sports, University of Ljubljana. The room temperature was between 20 and 24°C, and the humidity was between 33 and 65%. For the ET, a motorized treadmill (Cosmed, h/p/cosmos sports, and medical GmbH, Nussdorf-Traunstein, Germany) was used. The exercise protocol was adapted from Riggs et al. (37).

**Figure 1:**
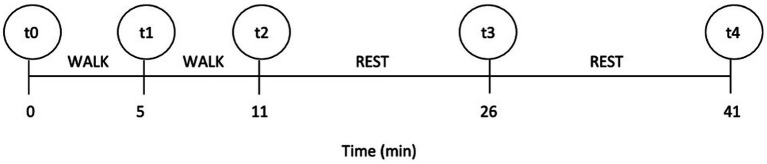
The timeline of the testing protocol; t0–t4 indicate the measuring points at which rectal temperature and heart rate were measured.

The test was standardized for all dogs and consisted of two successive parts. Before starting the experiment, all dogs freely familiarized themselves with the environment and operators for 15 min. Prior to the start of ET (at the measuring point t0), each dog’s baseline RT (T0) and heart rate (HR) were measured. All dogs that participated had never walked on a treadmill before. Fitness level of all dogs in the study was similar and impaired due to BOAS. All dogs participating in ET were ready to walk on a treadmill without prior habituation. One of the examiners led dogs onto the treadmill, where they were tethered to the examiner with a leash and harness. Owners stood in front of the treadmill to encourage the dogs. The second examiner was at the treadmill control panel in case the treadmill had to be stopped promptly. In the first part, the dog had to walk for 5 min at a speed of 2.5 km/h and an inclination of 0%. After 5 min, we stopped the treadmill to perform the measurements (t1) of RT and HR; we then continued with the second part. In the second part, the dog walked for 5 min at a speed of 2.5 km/h and an inclination of 5%. Immediately after exercise (t2) ended, RT and HR were measured again. After ET, the dogs rested for 30 min to recover. In the recovery, RT and HR were measured 15 min (t3) and 30 min (t4) after the cessation of ET. The testing protocol with the timeline depicting ET and recovery is shown in Figure 1.

HR was measured manually on the femoral artery, and RT was measured with a digital thermometer (Microlife, Microlife AG, Widnau, Switzerland).

ET was stopped immediately if exhaustion, labored breathing, or cyanosis were noted.

The same ET protocol was performed 6–9 months after the surgery.

On the same day, after ET had been performed, the dogs underwent the surgical treatment of BOAS. Before surgery, the upper airways were examined by the same person with a rigid and flexible endoscope under general anesthesia, as previously described by Erjavec et al. (36). Confirmation of BOAS and evaluation of the severity of pharyngolaryngeal obstruction were performed as described by Lilja-Maula et al. (38). During the surgical procedure, the same person corrected stenotic nares and an elongated and thickened soft palate in all dogs. For stenotic nares, a vertical wedge rhinoplasty was performed (1) by excising a portion of the ala nasi with a No. 11 scalpel blade and suturing it with Glycomer 631 synthetic absorbable monofilament suture material (Biosyn 4/0, Covidien, Dublin, Ireland). The elongated and thickened soft palate was corrected with a folded flap palatoplasty (40) in which a portion of the oropharyngeal mucosa and underlying soft tissue was excised with a Surgitron Radiolase II radio-surgical device (Ellman International, Inc., Hicksville, NY, United States) and sutured with single interrupted sutures using Glycomer 631synthetic absorbable monofilament suture material (Biosyn 4/0, Covidien, Dublin, Ireland).

### 2.3. Statistical analysis

Data were analyzed with commercial software (IBM SPSS 28, Chicago, Illinois, United States). The Shapiro–Wilk test was performed to determine the distribution of data. Normally distributed data are reported as means ± standard deviation (SD), whereas non-normally distributed as a median and interquartile range (IQR – 25th to 75th percentile), respectively.

Differences of the corresponding RT between the definite point (t1–t4) and the baseline (t0) were calculated and expressed as delta temperature (ΔT), as follows: ΔT1: T1-T0, ΔT2: T2-T0, ΔT3: T3-T0 ΔT4: T4-T0.

According to data distribution, non-parametric tests were used to evaluate differences in HR, ΔT, and the sum of the individual sets of clinical signs (respiratory, gastrointestinal, sleep disorders, and exercise intolerance), and parametric tests to evaluate the differences in RT. Accordingly, Friedman analysis with multiple comparisons and Bonferroni adjustments (HR) or a repeated measures ANOVA with Bonferroni adjustments (RT) was used to test for statistically significant differences in HR and RT between the different measuring points before and after the surgery. At each measuring point, a Wilcoxon signed-rank test (HR, ΔT, the sum of the individual sets of clinical signs) or a paired sample *t*-test (RT) was used to compare the parameters before and after the surgery.

A *p-*value ≤ 0.05 was considered statistically significant.

## 3. Results

The baseline characteristics of the evaluated dogs are summarized in Table 1.

**Table 1:** Baseline characteristics of the included dogs.

Number	Sex (male/female)	Age (months) Mean ± SD	Body weight (kg) Mean ± SD	BCS Median (IQR)	Baseline HR (bpm) Median (IQR)
13	10/3	32.0 ± 16.0	11.30 ± 2.08	3.0 (3.0–3.3)	116 (108–122)

BCS, body condition score; baseline HR, resting heart rate before exercise testing; bpm, beats per minute; IQR, interquartile range (25th to 75th percentile); SD, standard deviation.

Exercise test had to be stopped before terminating the protocol only in one dog, in both settings, i.e., before and after the surgery. Before the surgery, the test had to be stopped after 3 min of ET because of labored breathing and vomiting; after the surgery, ET was halted 1 min after the inclination part because of increased inspiratory effort. For this French bulldog, the HR and RT measurements were not included in the statistical analysis.

**Figure 2:**
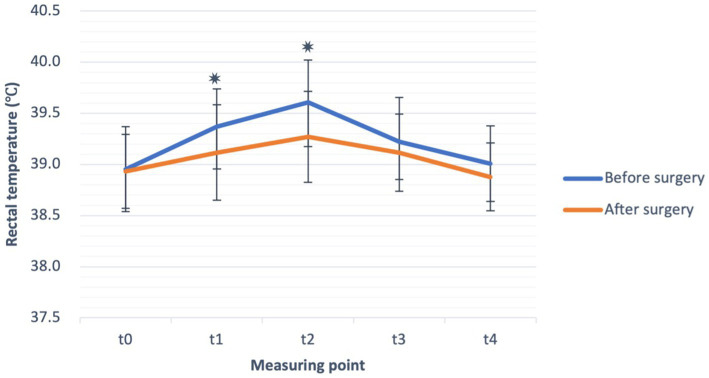
Rectal temperature (°C) (mean ± SD) before exercise (t0), during exercise test (t1, t2) and in the recovery (t3, t4), before (blue line), and after the surgery (orange line) in French bulldogs (*n* = 13). *p ≤ 0.05, paired sample *t*-test.

No significant difference was found in baseline RT, measured at time point t0 (T0) in dogs before and after the surgery. At the start of ET and during exercise, RT started to rise in both settings, i.e., before and after the surgery, as shown in Figure 2. Compared to the baseline, RT increased significantly at t1 (*p* = 0.006) and t2 (*p* = 0.001) before the surgery. After the surgery, RT increased significantly (*p* = 0.006) only at t2 compared to t0. The absolute value of RT, measured at both t1 (*p* = 0.004) and t2 (*p* < 0.001), was significantly lower after than before the surgical treatment (Figure 2). When comparing the absolute values of RT in the recovery (measuring points t3 and t4) before and after the surgery, although a trend of lower T3 and T4 after the surgery was observed, it did not reach statistical significance.

When comparing differences of RT between t1–t4 and t0 (ΔT) before and after the surgery, significantly (*p* = 0.033) lower ΔT2 after the surgical treatment (Table 2), similarly as for the absolute values of RT, was observed.

**Table 2:** Temperature increments (ΔT) during exercise and in recovery are expressed as the temperature difference between a particular measurement point (t1–t4) and the baseline temperature (T0) at the measurement point t0 before and after the surgery.

Temperature increment (ΔT)	Before surgery Median; IQR	After surgery Median; IQR	*p*-value
ΔT1 (°C)	0.40; (0.15–0.60)	0.10; (0.00–0.35)	0.067
ΔT2 (°C)	0.60; (0.35–0.90)	0.30; (0.10–0.45)	0.033*
ΔT3 (°C)	0.20; (−0.10–0.60)	0.20; (0.00–0.35)	0.875
ΔT4 (°C)	0.10; (−0.35–0.20)	0.00; (−0.25–0.10)	0.633

IQR, interquartile range (25th to 75th percentile); for the description of ΔT, see Materials and methods section (statistical analysis). *p ≤ 0.05, Wilcoxon signed-rank test.

**Figure 3:**
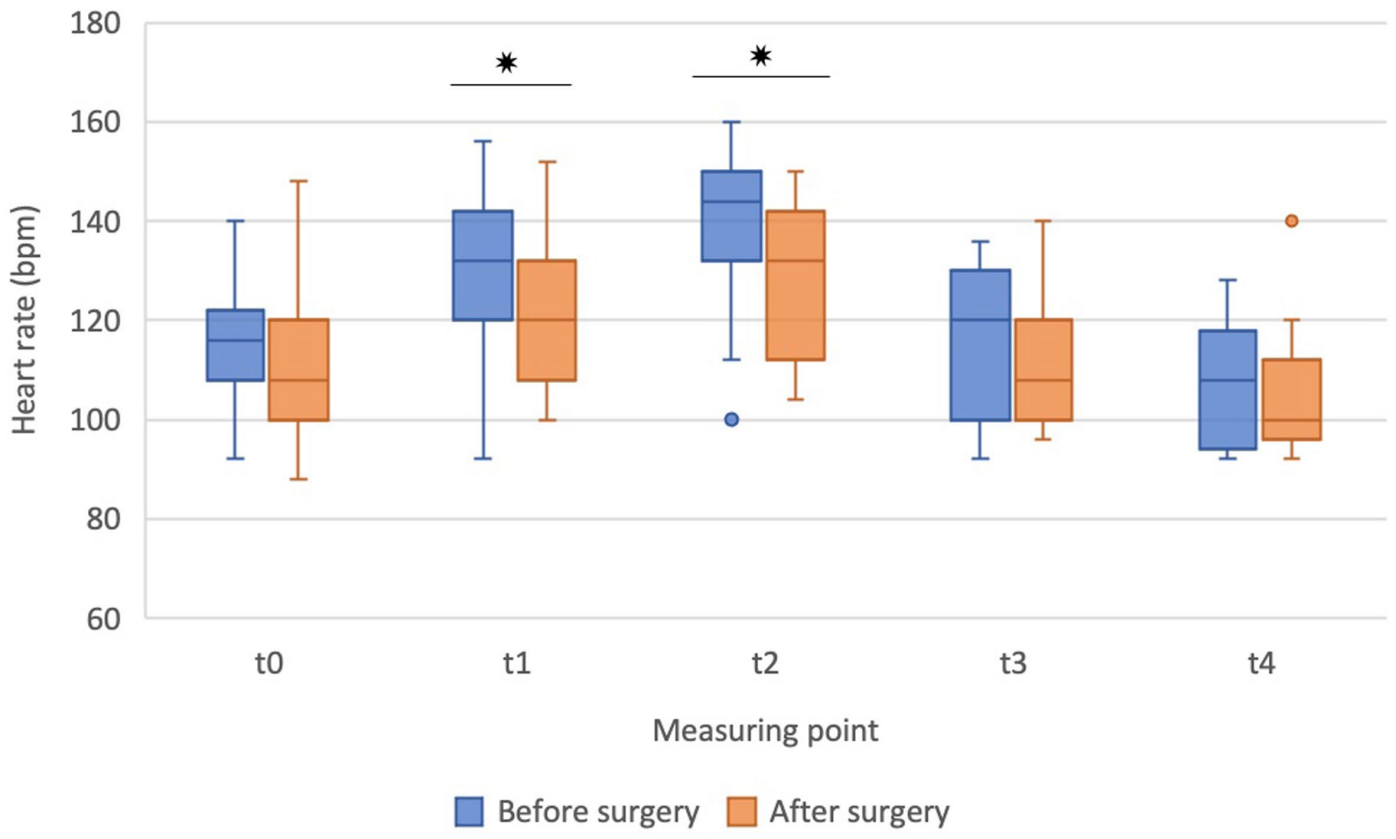
The heart rate before (t0), during exercise (t1, t2), and in the recovery (t3, t4) before the surgery (blue boxes) and after the surgery (orange boxes) in French bulldogs (*n* = 13). Data are presented as box plots. *p ≤ 0.05, Wilcoxon signed-rank test. Bpm beats per minute.

During ET, the HR increased in both settings, i.e., before and after the surgery, and was significantly higher in points t1 and t2 compared to baseline (t0) (Figure 3) but returned to baseline value soon after the cessation of exercise, i.e., at points t3 and t4. Before the surgery, HR increased significantly at t1 (*p* = 0.030) and t2 (*p* = 0.001) compared to baseline (t0). After the surgery, HR also increased significantly at t1 (*p* = 0.018) and t2 (*p* < 0.001) compared to baseline (t0). However, the HR at points t1 (*p* = 0.020) and t2 (*p* = 0.011) were significantly lower after the surgical treatment (Figure 3). Similarly, as for the RT in the recovery, there was a trend of lower HR after the surgery.

On upper airway examination to assess the severity of pharyngolaryngeal obstruction, 8/13 dogs were classified as having grade 2 obstruction and 5/13 dogs were classified as having grade 3 obstruction.

The analysis of the owner questionnaires (Table 3) showed that exercise intolerance and respiratory problems were the main problems of the dogs before the surgery, followed by sleep and gastrointestinal disorders. Six to nine months after the surgery, when owners reassessed clinical signs, a statistically significant improvement in all clinical signs, with the greatest improvement in exercise tolerance after the surgery was observed.

**Table 3:** Clinical signs [median, interquartile range (IQR)] were evaluated by the owners in the “owner” questionnaire using a five-point scale before and after the surgery.

	Respiratory signs Median (IQR)	Gastrointestinal signs Median (IQR)	Exercise intolerance Median (IQR)	Sleep disorders Median (IQR)
Before surgery	2.4 (2.1–3.4)	1.8 (1.2–2.9)	3.8 (3.5–4.2)	2.0 (1.4–2.1)
After surgery	1.5 (1.45–2.5)*	1.3 (1.1–1.8)*	2.5 (1.9–3.4)*	1.1 (1.0–1.6)*

IQR, interquartile range (25th to 75th percentile). *p ≤ 0.05, Wilcoxon signed-rank test; 1.0 means the clinical sign was not present, and 5.0 means the clinical sign was always present.

## 4. Discussion

The main finding of our study is that surgical correction of BOAS in French bulldogs positively impacts their body temperature profile during exercise, which indicates improved thermoregulatory response. To the best of our knowledge, the present study is the first to compare the thermoregulatory response, assessed by measuring the RT during exercise and in the recovery of French bulldogs as representatives of extremely brachycephalic breeds before and after surgical treatment of BOAS.

Exercise testing (to submaximal or lower intensity) in veterinary medicine has been described in canine cardiology in dogs with congestive heart disease and mitral valve disease to test the effect of treatment (41–44). In dogs with BOAS, exercise testing has been applied in English bulldogs, pugs, and French bulldogs to assess the severity of BOAS (37, 38, 45–47). Nevertheless, in BOAS, the exercise intensity is far below submaximal: e.g., the workload used in the study of Wall et al. (42) and in Mach et al. (47) was set far below submaximal which is between 60 and 80% of HRmax (i.e., HR between 180 and 240 bpm) (48). The exercise testing protocol in BOAS was designed to ensure a minimal workload inducing a 40% increase in the HR compared to the resting HR (42, 47). In the French bulldogs diagnosed with BOAS+ included in our study and requiring surgical treatment, their owners reported that the dogs had problems with exercise intolerance, as even a 10-min walk in the summer was a problem for them. Clinical signs confirmed in BOAS+ patients were respiratory noises (stridor, stertor), increased respiratory effort, abdominal breathing, exercise intolerance, overheating, regurgitation, vomiting, and sleep disorders. In accordance with the reports of the owners and clinical examination findings, a testing protocol from Riggs et al. (37), who used a 5-min walk test and a 3-min trot test (37), was adapted. In ET of the present study, the respiratory and thermoregulatory limitations of the French bulldogs that participated in the study using a 5-min walk test but modified the second part, i.e., the 3-min trot test to a 5-min walk test with a 5% inclination were considered. As some of the dogs included in the present study presented with moderate dyspnoea and were unable to exercise, i.e., could not trot with their owners, it was considered that trotting might pose a risk for the dogs to develop hyperthermia and therefore they would have to be excluded from the study. During ET, the upper airway function of the dogs was challenged, resulting in open-mouth breathing and showing worsening of previously described clinical signs of BOAS, such as louder upper respiratory noises (stridor, stertor), dyspnoea and tachypnoea (19, 37), implying that exercise intensity was sufficient to challenge the thermoregulatory response. In addition, the overall cardiorespiratory status of the included dogs was rather low, as evident from their resting HR, the owner questionnaire score, and the medical history provided by their owners. As RT in dogs increased during exercise, implying increased heat production, it is believed that the protocol was appropriate to induce a measurable thermoregulatory response. Moreover, both RT and HR significantly increased during the testing. In daily life, especially during the summer months when ambient temperatures exceed 24°C, often reaching values between 30°C and 40°C, these exercise-induced changes in RT would be more pronounced; it is believed that the effect and benefits of surgical correction of the upper airways would correspondingly be more pronounced.

The use of motorized treadmills without any familiarization period may make dogs unwilling to walk or run on them (49). In the present study, it was observed that all participating dogs were willing to walk on the treadmill without any familiarization period, even if they had never walked on a treadmill before, although French bulldogs and other brachycephalic breeds are known to be lazy at times. One reason may be that owners were present in the laboratory during the treadmill test and stood in front of their dogs while they were walking. In addition, owners constantly encouraged and motivated their dogs to walk toward them during the ET. Similar results have been described in other studies (40, 50). Moreover, the speed in this ET was relatively low, and the dogs could easily adapt to it.

Exercise can induce a 200% rise in body heat production in healthy dogs (31). With the presence of anatomical aberrations, such as stenotic nares, aberrant nasal conchae, hypertrophic mucosa, mucosal contact points, and stenotic laryngeal cartilages that impair normal thermoregulatory capabilities, heat production in brachycephalic dogs is expected to be even higher; therefore, monitoring body temperature is necessary during exercise testing in brachycephalic dogs (2, 3, 16, 20). In our study, the RT of French bulldogs before the surgery increased significantly during ET. The present study showed that the mean increase of RT in dogs before surgery after the end of ET was 0.60°C, which is consistent with the study of Aromaa et al. (46), in which the mean increase in body temperature after submaximal exercise in French bulldogs was also 0.60°C, although dogs in their study were subjected to more intensive exercise (1,000 m walking or trotting). For English bulldogs, Lilja-Maula et al. (38) reported the mean increase in body temperature after the end of submaximal exercise of 0.43°C, and Mach et al. (47) reported that the mean increase in body temperature after submaximal exercise in pugs was 0.5°C.

As expected, RT also increased in the second setting of experiments, i.e., after surgery; however, the increase in RT was significantly smaller. Compared to the increase of RT before the surgery (0.60°C), the mean increase after the surgery was much lower, only 0.30°C, indicating a beneficial effect of surgery. The same effect was also observed during recovery. The goal of BOAS corrective surgery is to reduce airway resistance (1). Poiseuille’s law states that a 50% reduction of the radius induces a 16-fold increase in resistance to flow (2, 51). Therefore, with the widening of ala nasi and shortening and thinning of the soft palate, soft tissue obstruction and, thus, resistance decrease. Because the main heat exchange during resting occurs at the nasal epithelial lining of the ventral nasal concha, widening the nostrils could allow more air to reach the epithelium (52), thus increasing the evaporative heat exchange despite the anatomically smaller evaporative area. This could improve thermoregulation during rest at higher ambient temperatures and during sleep, meaning that dogs could cool down more efficiently and recover faster after activities and during the summer months. When exposed to exercise or high environmental temperatures, ventilation occurs through the mouth and nose, and heat loss is additionally achieved by panting (18, 19).

During exercise, increased HR is the main determinant of cardiac output, oxygen uptake, and an index of cardiovascular workload (53, 54). In accordance with previous studies conducted on brachycephalic dogs during exercise (38, 46, 47), the present study has also shown a significant increase in HR during ET. However, the rise of HR was significantly smaller after the surgery than before. Moreover, at the end of ET, the HR was lower after than before the surgery, both indicating a potential effect of surgical correction on hemodynamic variables affecting cardiac output, determining blood flow, and, indirectly, heat exchange. Accordingly, a similar dynamic of RT and HR was observed during exercise, at least at some points, although statistical correlations between these two parameters were not determined. Finally, differences in exercise-induced increase of HR before and after the surgery imply higher oxygen consumption before the surgery (55). Higher requirements for oxygen in dogs before the surgery are caused by the narrowing of soft tissues of the upper airway and the subsequential obstruction they create, increasing the resistance to airflow (2, 3), implying an additional burden on respiratory muscles demanding more oxygen. Surgical intervention decreases the obstruction and resistance to flow, alleviating symptoms and decreasing the work of breathing (1). In addition, improved ventilation enables a more efficient thermoregulatory response through evaporative heat loss due to increased evaporative surface.

Some might argue that decreased HR in the second setting of experiments (i.e., after the surgery) might be since dogs were already familiar with the ET and treadmill and thus exposed to less anxiety and psychological stress. However, this option seems less probable as, firstly, six to nine months have passed from the first testing to the second testing after surgery, and, secondly, and even more significantly, similar changes were found in the dynamics of RT that is not affected by psychological stress as the HR is.

During the measurements, the dogs stopped exercising, which is a study limitation. The measurement of HR was done manually and intermittently, potentially addressing the objectivity and relevance of the method used. A heart rate monitor would enable more objective and continuous measurements of HR; this is also true for the RT assessment. Nevertheless, since all dogs were exposed to the same interrupted protocol, and the time to execute manual measurements were similar in all dogs, the potential bias of the study was substantially reduced. Perhaps protocol disruption could also be avoided and stress minimized by measuring skin temperature with an infrared skin sensor (IR), as described in a study in horses (56), but this method is not a reliable alternative for measuring thermoregulatory response because it does not accurately estimate changes in body core temperature. An alternative to non-invasive temperature measurement is infrared thermography. Non-contact infrared thermometers can be used to measure auricular and ocular temperature. Auricular temperature can be used to estimate body core temperature in healthy dogs under controlled conditions and ambient temperatures, but a conversion factor must be applied to these measurements to obtain the correct estimate (57, 58). Measuring ocular temperature with non-contact infrared thermometers may underestimate body temperature in hyperthermic dogs (59). Accordingly, it is believed that the measurement of RT applied in the present study could be regarded as a potentially reliable indicator of body core temperature.

A similar trend of RT and HR changes during ET after the surgery was observed in the present study. Thermoregulation during exercise, additionally to exercise *per se*, increases the HR, one of the main determinants of cardiac output (20, 53). Respectively, surgery can also be proposed to alleviate cardiac work, thus improving clinical signs.

The questionnaire results showed that the clinical signs before surgery, such as breathing problems, exercise intolerance, sleep disorders, and gastrointestinal symptoms that owners observed in their animals, were consistent with the results of several previous studies (36, 60). The main problems observed before surgery were exercise intolerance and respiratory problems. After the surgery, the owners reported improvements in all categories of clinical signs. The most significant improvement was observed in exercise tolerance and respiratory problems: the dogs could walk longer, endured more physical exertion, required less time to recover after activity, and showed less respiratory effort during physical activity. Sleep and gastrointestinal disorders were almost completely resolved after the surgery. The improvements observed in the dogs after surgery show that the quality of life of these dogs has improved. Moreover, the owners’ quality of life has also improved: they are less worried about whether their dog can go for a daily walk, how it recovers after activity, and how it can tolerate the higher temperatures in the summer months.

### 4.1. Limitations

The authors acknowledge several limitations of the study. First, the sample size was rather small. Since the dogs were all the same breed, and their body mass and cardiovascular fitness were rather homogenous, it is believed that enough dogs were included for a pilot study.

Another issue is manual and intermittent measurement of HR and RT, during which dogs stopped exercising. The dogs were only familiarized with the environment and operators before baseline testing but not with the treadmill, which is also a limitation of the present study.

## 5. Conclusion

Our pilot study’s results have shown that surgical management to reduce increased upper airway obstruction in French bulldogs improved both the RT and the HR profile during different time points of treadmill exercise and its recovery, implying improved thermoregulation. Accordingly, surgery should be advised to owners of dogs exhibiting BOAS with prominent clinical signs; more studies, including larger sample size and other breeds with extreme brachycephaly, are warranted to evaluate the effect of surgical treatment in brachycephalic breeds more objectively.

## Data availability statement

The original contributions presented in the study are included in the article/Supplementary material, further inquiries can be directed to the corresponding author.

## Ethics statement

The animal studies were approved by Animals in Experiments Welfare Commission of the Veterinary Faculty, University of Ljubljana, approval number 18-3/2022-1. All procedures were conducted in accordance with the Slovenian Animal Protection Act (The Official Gazette of the Republic of Slovenia, 43/2007). The studies were conducted in accordance with the local legislation and institutional requirements. Written informed consent was obtained from the owners for the participation of their animals in this study.

## Author contributions

ŽŽ, ANS, HL, JV, and VE contributed to the conceptualization of the study, data curation, and drafting of the initial manuscript and approved the submitted version of the manuscript. ŽŽ and VE coordinated the clinical conduction (i.e., measurements and surgery) of the research. JV supervised the exercise testing. ANS contributed to data handling and statistical analysis. HL critically revised the manuscript for important physiological content and data analysis. All authors contributed to the article and approved the submitted version.
