# Supplementary material for: The effect of the surgical treatment of brachycephalic obstructive airway syndrome on the thermoregulatory response to exercise in French bulldogs: a pilot study
Source: Front Vet Sci. 2023 Oct 12;10:1229687. doi: 10.3389/fvets.2023.1229687 (PMC10601647; doi:10.3389/fvets.2023.1229687)
Supplement: Supplementary file 1 [file Data_Sheet_1.pdf]

Supplementary Material

The effect of surgical treatment of Brachycephalic Obstructive Airway Syndrome on thermoregulatory response to exercise in French bulldogs: A pilot study

Žiga Žgank, Alenka Nemec Svete, Helena Lenasi, Janez Vodičar, Erjavec Vladimira*

*** Correspondence:** vladimira.erjavec@vf.uni-lj.si

1 Supplementary Data

Pre and postoperative owner questionaire on condition of the dog with brachycephalic syndrome

Which breed is your dog?

- English Bulldog
- French bulldog
- Pug
- Pekingese
- Boston terrier
- Shih-tzu
- Cavalier king Charles spaniel
- Boxer
- Other: ________________

Age of you animal?

_____________ years, _________months

Body weight or BMI (done by your veterinarian)

____

What sex is your animal?

- Male
- Female

Is your dog castrated/neutered?

YES NO

At what age did your animal show signs of brachycephalic syndrome (loud, labored breathing, digestive problems (frequent gagging, retching, vomiting), sleeping problems, snoring, excercise intolerance and inability to cool down at higher ambient temperatures)?

____

Has your dog had a surgery for brachicephalic syndrom? If yes, please atate when, at what age? ________________________

Several claims are listed. Please circle those that apply to medical condition and the behavior of your dog. The scale is from 1 (not true or never) to 5 (completely true

or permanently/always). An additional option is don't now.

**Breathing**	**Before surgery**	**After surgery**
- Breathes loudly at rest	1 2 3 4 5 don’t know	1 2 3 4 5 don’t know
- Breathes loudly when moving	1 2 3 4 5 don’t know	1 2 3 4 5 don’t know
- Breathes very fast	1 2 3 4 5 don’t know	1 2 3 4 5 don’t know
- Labored and loud inspiration	1 2 3 4 5 don’t know	1 2 3 4 5 don’t know
- Has longer pauses while breathing (could also be during sleeping)	1 2 3 4 5 don’t know	1 2 3 4 5 don’t know
- Breathing noises and crackles heard	1 2 3 4 5 don’t know	1 2 3 4 5 don’t know
- It coughes often	1 2 3 4 5 don’t know	1 2 3 4 5 don’t know
- It sneezes often	1 2 3 4 5 don’t know	1 2 3 4 5 don’t know
- Has prominent nasal discharge (watery)	1 2 3 4 5 don’t know	1 2 3 4 5 don’t know
- Has good sense of smell	1 2 3 4 5 don’t know	1 2 3 4 5 don’t know
- Almost entirely breathes trough mouth	1 2 3 4 5 don’t know	1 2 3 4 5 don’t now
- Breathes with his head & neck extended	1 2 3 4 5 don’t know	1 2 3 4 5 don’t know
- It is suffocating	1 2 3 4 5 don’t know	1 2 3 4 5 don’t know
- Ever lost its consciousness	1 2 3 4 5 don’t know	1 2 3 4 5 don’t know
- Difficult breathing on walks	1 2 3 4 5 don’t know	1 2 3 4 5 don’t know
- Difficult breathing after walk	1 2 3 4 5 don’t know	1 2 3 4 5 don’t know
- Short of breath in afternoon	1 2 3 4 5 don’t know	1 2 3 4 5 don’t know
- Very difficult breathing in summer	1 2 3 4 5 don’t know	1 2 3 4 5 don’t know
- Difficult breathing when excited	1 2 3 4 5 don’t know	1 2 3 4 5 don’t know

How often does "reverse sneezing" occur or successive, loud inhalations and exhalations lasting several seconds (it looks like the animal is about to suffocate)?

- Occurs several times a day
- Occurs every day
- Occurs at least once a week
- Occurs at least once a month
- Occurs once a year
- Does not occure

Sleeping

- Snores	1 2 3 4 5 don't know	1 2 3 4 5 don't know
- Sleeps with head up.	1 2 3 4 5 don't know	1 2 3 4 5 don't know
- Sleeps or napps in sitting position	1 2 3 4 5 don't know	1 2 3 4 5 don't know
- Sleeps with open mouth	1 2 3 4 5 don't know	1 2 3 4 5 don't know
- During sleeping stopps breathing for few moments	1 2 3 4 5 don't know	1 2 3 4 5 don't know
- Starts gagging and retching during sleep	1 2 3 4 5 don't know	1 2 3 4 5 don't know
- Sleeps with a toy or sth else in the mouth	1 2 3 4 5 don't know	1 2 3 4 5 don't know
- It can hardly ever sleep	1 2 3 4 5 don't know	1 2 3 4 5 don't know
- It never sleeps	1 2 3 4 5 don't know	1 2 3 4 5 don't know

Gastrointestinal disorders

Please circle the choosen answer **YES** or **No.**

- Dog vomits. YES NO

- Dog vomits by tensing the abdominal muscles and diaphragm, has an extended neck and head turned downward.

YES NO

- Dog vomits without straining, it stretches its head and the contents come out of its mouth.

YES NO

- Dog gaggs and then vomits only foam and saliva.

YES NO

Please claim how ofter does your dog vomit __________________ daily/weekly/monthly/yearly.

Several claims are listed. Please circle those that apply to medical condition
and the behavior of your dog. The scale is from 1 (not true or never) to 5 (completely true

or permanently/always). An additional option is don't now.

**Gastrointestinal problems**	**Before surgery**	**After surgery**
- Vomits immmediatelly after a meal	1 2 3 4 5 don’t know	1 2 3 4 5 don’t know
- Vomits during or after a walk	1 2 3 4 5 don’t know	1 2 3 4 5 don’t know
- Vomits during or after excitement	1 2 3 4 5 don’t know	1 2 3 4 5 don’t know
- Vomits foam, saliva, and water	1 2 3 4 5 don’t know	1 2 3 4 5 don’t know
- Vomits food	1 2 3 4 5 don’t know	1 2 3 4 5 don’t know
- Drools excesively	1 2 3 4 5 don’t know	1 2 3 4 5 don’t know
- Swallow vomited contents	1 2 3 4 5 don’t know	1 2 3 4 5 don’t know
- Often swallows without eating	1 2 3 4 5 don’t know	1 2 3 4 5 don’t know
- Is on medication for gastritis	1 2 3 4 5 don’t know	1 2 3 4 5 don’t know
- Has sensitive digestion	1 2 3 4 5 don’t know	1 2 3 4 5 don’t know

**Behaviour and physical activity**	**Before surgery**	**After surgery**
- Gets most tired in summer	1 2 3 4 5 don't know	1 2 3 4 5 don't know
- Stops and rests on walks	1 2 3 4 5 don't know	1 2 3 4 5 don't know
- Drinks a lot of water	1 2 3 4 5 don't know	1 2 3 4 5 don't know
- It overheats often	1 2 3 4 5 don’t know	1 2 3 4 5 don't know
- Stands with front legs wide apart	1 2 3 4 5 don’t know	1 2 3 4 5 don't know
- Gets tired much earlier during the play than others dogs	1 2 3 4 5 don’t know	1 2 3 4 5 don’t know
- 10-minute walk in summer is a lot of effort for a dog	1 2 3 4 5 don’t know	1 2 3 4 5 don’t know
- 20-minute walk in summer Is a lot of effort for a dog?	1 2 3 4 5 don’t know	1 2 3 4 5 don’t know
- 30-minute walk in summer is a lot of effort for a dog	1 2 3 4 5 don’t know	1 2 3 4 5 don’t know
- 1-hour walk in summer is a lot of effort for a dog	1 2 3 4 5 don’t know	1 2 3 4 5 don’t know

How long does it take to recover from a play or walk in summer and in winter?

In the summer	In winter
- At least 5 minutes	- At least 5 minutes
- Up to 15 minutes	- Up to 15 minutes
- Up to 30 minutes	- Up to 30 minutes
- Up to 1 hour	- Up to 1 hour

At what outside temperature do breathing problems begin?

- It always has problems, regardless of the outside temperature
- At T over 10 ˚C
- At T over 15 ˚C
- At T over 18 ˚C
- At T over 20 ˚C
- At T over 23 ˚C
- At T over 26 ˚C
- At T over 30 ˚C

Which problem is most pronounced in your dog?

- Excercise intolerance
- Breathing difficulties
- Overheating and inability to cool down
- Difficulties while sleeping
- Difficulties while eating
